# Searching for phenotypic causal networks involving complex traits: an application to European quail

**DOI:** 10.1186/1297-9686-43-37

**Published:** 2011-11-02

**Authors:** Bruno D Valente, Guilherme JM Rosa, Martinho A Silva, Rafael B Teixeira, Robledo A Torres

**Affiliations:** 1 Department of Animal Sciences, Federal University of Minas Gerais, 30123-970, Brazil; 2 Department of Animal Sciences, University of Wisconsin, Madison, Wisconsin USA; 3 Department of Biostatistics and Medical Informatics, University of Wisconsin, Madison, Wisconsin USA; 4 Department of Animal Sciences, Federal University of Viçosa, 36570-000, Brazil

## Abstract

**Background:**

Structural equation models (SEM) are used to model multiple traits and the casual links among them. The number of different causal structures that can be used to fit a SEM is typically very large, even when only a few traits are studied. In recent applications of SEM in quantitative genetics mixed model settings, causal structures were pre-selected based on prior beliefs alone. Alternatively, there are algorithms that search for structures that are compatible with the joint distribution of the data. However, such a search cannot be performed directly on the joint distribution of the phenotypes since causal relationships are possibly masked by genetic covariances. In this context, the application of the Inductive Causation (IC) algorithm to the joint distribution of phenotypes conditional to unobservable genetic effects has been proposed.

**Methods:**

Here, we applied this approach to five traits in European quail: birth weight (BW), weight at 35 days of age (W35), age at first egg (AFE), average egg weight from 77 to 110 days of age (AEW), and number of eggs laid in the same period (NE). We have focused the discussion on the challenges and difficulties resulting from applying this method to field data. Statistical decisions regarding partial correlations were based on different Highest Posterior Density (HPD) interval contents and models based on the selected causal structures were compared using the Deviance Information Criterion (DIC). In addition, we used temporal information to perform additional edge orienting, overriding the algorithm output when necessary.

**Results:**

As a result, the final causal structure consisted of two separated substructures: BW→AEW and W35→AFE→NE, where an arrow represents a direct effect. Comparison between a SEM with the selected structure and a Multiple Trait Animal Model using DIC indicated that the SEM is more plausible.

**Conclusions:**

Coupling prior knowledge with the output provided by the IC algorithm allowed further learning regarding phenotypic causal structures when compared to standard mixed effects SEM applications.

## Background

Structural equation models or SEM [1,2] are used to model multiple traits and functional links among them, which may be interpreted as causal relationships. These models were adapted for the context of quantitative genetics mixed models by [3], and henceforth applied and extended by a number of authors [4-11].

Fitting SEM requires choosing a causal structure *a priori*. This structure describes qualitatively the causal relationships among traits by determining the subset of traits that imposes causal influence on each phenotype studied. By fitting a SEM, it is possible then to infer the magnitude of each causal relationship pertaining to the causal structure, which is quantified by model parameters called structural coefficients. However, choosing the causal structure may be cumbersome, given the typically very large space of possible causal hypotheses, even when only a few traits are studied. The choice of causal structures for the aforementioned SEM applications that followed the work of [3] were performed on the basis of prior beliefs, resulting in poor exploration of structures spaces.

Methodologies such as the IC algorithm [12,13] make it possible to search for recursive causal structures that are compatible with the joint probability distribution of the variables considered. Therefore, applying these methodologies allows the selection of causal structures without relying on prior knowledge alone. Nonetheless, such algorithms are constructed based on specific assumptions regarding the data, such as the causal sufficiency assumption (for more details, see [12,14]). Under this assumption, the residuals of the SEM for which the causal structure will be chosen are regarded as independent between traits. This construction is necessary to establish the connection between the selected causal structures and the joint probability distribution under study, such that d-separations [12,14] in causal structures among traits are reflected as null partial correlations. Under this scenario, the IC algorithm takes a correlation matrix as input and searches for causal structures that are capable of producing that matrix, with its conditional dependencies and independencies. However, multiple phenotypes may present unobserved correlated genetic effects which confound such search, as discussed by Valente et al. [15]. When using mixed effects SEM to represent this scenario, this confounding may take place even if model residuals are regarded as independent. As an alternative, Valente et al. [15] proposed a methodology which couples Bayesian model fitting and the application of the IC algorithm to the joint distribution of phenotypes conditional on the genetic effects.

With the purpose of validating and illustrating their method, Valente et al. [15] applied it to simulated data based on different scenarios. Here, we present the first application of such methodology to a real data set, by exploring the space of causal structures among five productive and reproductive traits in European quail. The discussion is focused on the challenges and benefits resulting from applying this method to field data, as well as on proposing approaches to overcome such challenges.

## Methods

### Data

The data refer to 849 female European quail (*Coturnix coturnix coturnix*) from six distinguished hatch seasons. The birds were raised in an experimental station, with *ad libitum *access to water and 2, 900 kcal/kg and 28% crude protein diet. They were kept on the floor until 35 days of age, and then transferred to individual cages, and provided a laying diet henceforth. Five traits were analyzed: birth weight (BW), weight at 35 days of age (W35), age at first egg (AFE), average egg weight from 77 to 110 days of age (AEW), and number of eggs laid in the same period (NE). Measurements for all five traits were available for every bird, with no missing data. Means and standard deviations for each trait are presented in Table 1. Additionally, the analysis considered pedigree information, containing 10, 680 individuals.

**Table 1:** Mean and standard deviation (SD) for each trait

**Trait**^**a**^	Mean	SD
BW	10.06	0.94
W35	262.30	25.13
AFE	53.32	10.14
AEW	13.58	1.29
NE	29.98	7.42

^a ^BW = birth weight (g); W35 = weight at 35 days (g); AFE = age at first egg (days); AEW = average egg weight from 77 to 110 days (g); NE = number of eggs laid from 77 to 110 days

### Structural equation models

The SEM used to fit the data may be represented as [3,15]:

(1) $$y=\left(\text{Λ}⊗I_{n}\right)y+X\beta +Zu+e,$$

with the joint distribution of vectors **u **and **e **as:

(2) $$\left[\begin{array}{c} u\\ e \end{array}\right]\sim N\left\{\left[\begin{array}{c} 0\\ 0 \end{array}\right],\left[\begin{array}{cc} G_{0}⊗A & 0\\ 0 & \Psi_{0}⊗I_{n} \end{array}\right]\right\},$$

where **y**, **u **and **e **are, respectively, vectors of phenotypic records, additive genetic effects and model residuals for *t *traits, sorted by trait and subject within trait; **β **is a vector containing the (fixed) effects of hatch season for each trait; **X **and **Z **are incidence matrices relating effects in **β **and **u **to **y**; **Λ **is a (*t *× *t*) matrix with zeroes on the diagonal and with structural coefficients or zeroes on the off-diagonal (the causal structure defines which entries contain free parameters and which entries are constrained to 0); **G**_0 _and **Ψ**_0 _are the additive genetic and residual covariance matrices, respectively; and **A **is the genetic relationship matrix, constructed from pedigree information. The model given by (1) may be rewritten as:

(3) $$\left[I_{tn}-\left(\text{Λ}⊗I_{n}\right)\right]y=X\beta +Zu+e,$$

such that the so-called reduced model is expressed as:

$$y={\left[I_{tn}-\left(\text{Λ}⊗I_{n}\right)\right]}^{-1}X\beta +{\left[I_{tn}-\left(\text{Λ}⊗I_{n}\right)\right]}^{-1}Zu+$$

(4) $${\left[I_{tn}-\left(\text{Λ}⊗I_{n}\right)\right]}^{-1}e.$$

Therefore,

$$p\left(y|\text{Λ},\beta ,u,\Psi_{0}\right)\sim N\left\{{\left[I_{tn}-\left(\text{Λ}⊗I_{n}\right)\right]}^{-1}\left(X\beta +Zu\right)\right),$$

(5) $$\left({\left[I_{tn}-\left(\text{Λ}⊗I_{n}\right)\right]}^{-1}\Psi {{\left[I_{tn}-\left(\text{Λ}⊗I_{n}\right)\right]}^{\prime }}^{-1}\right\},$$

where **Ψ **= **Ψ**_0_⊗**I**_*n*_.

### Recursive causal structure selection

Selection of causal structure was performed by following the methods presented by [15]. As mentioned by these authors, there are algorithms that search for recursive causal structures (i.e. causal structures with no cycles or feedback relationships between traits) assuming that conditional independencies in the joint probability distribution of the studied variables mirror d-separations in the causal structure (for more details, see [12,14-16]). One of such algorithms is the Inductive Causation (IC) algorithm, which is able to search, within typically vast causal structure spaces, for a class of minimal structures that are compatible with the conditional independencies carried by the joint distribution of the data. This class consists of statistically equivalent causal structures that impose the same set of stable conditional independencies in the joint distribution (i.e. they cannot be distinguished on the basis of data evidence) and may be represented by a partially oriented graph, i.e., a causal structure carrying directed and undirected edges, the latter representing causal connections with unspecified causal direction. The edges that are left undirected by the algorithm may present one direction or the other in different structures within the class, such that no direction results in causal cycles or further unshielded colliders (sub-structures consisting of unlinked vertices with a common child, such as *y_j_*→ *y_j'' _*← *y_j'_*, where *j*, *j'*, and *j'' *are indexes indicating three different phenotypic traits, and *y_j_*→ *y_j' _*indicates that *y_j _*directly affects *y_j'_*). The IC algorithm, when applied to a set **P **of *t *phenotypic traits, can be described as follows: 

Step 1. For each pair of phenotypic traits *y_j _*and *y_j' _*(*j *≠ *j' *= 1, 2, ..., *t*) in **P**, search for a set of traits **S**_*jj' *_such that *y_j _*is independent of *y_j' _*given **S**_*jj'*_. If *y_j _*and *y_j' _*are dependent for every possible **S**_*jj'*_, connect *y_j _*and *y_j' _*with an undirected edge. This step returns an undirected graph **U**.

Step 2. For each pair of non-adjacent traits *y_j _*and *y_j' _*with a common adjacent trait *y_j'' _*in **U **(i.e., *y_j _*- *y_j'' _*- *y_j'_*), search for a set **S**_*jj' *_containing *y_j'' _*such that *y_j _*is independent of *y_j' _*conditional on **S**_*jj'*_. If there is no such set, then add arrowheads pointing at *y_j'' _*(*y_j_*→ *y_j'' _*← *y_j'_*). Otherwise, continue.

Step 3. In the partially oriented graph returned by the previous step, orient as many undirected edges as possible in such a way that it does not result in new unshielded colliders or in cycles.

An important point to observe regarding the study of causal structures among phenotypic traits is that even if the residual covariance matrix is considered as diagonal, which is a consequence of the causal sufficiency assumption, unobserved correlated genetic effects act as sources of confounding [15,16]. Such feature damages the connection between causal structures and joint probabilities such that d-separations in the former are not expected to be reflected as conditional independencies in the latter. However, conditionally on the genetic effects, this connection is restored. Assessing this conditional probability distribution is possible since such effects can be 'controlled' based on a genetic distance matrix (e.g. a genetic relationship matrix). The conditional covariance matrix of **y **given **u **can be obtained by fitting a standard multiple trait animal model (MTAM, [17]) and obtaining the estimated residual covariance matrix, here represented by $R_{0}^{*}$. In some systems, other factors (e.g. correlated maternal effects) may also impose confounding in the search, and in these cases they should also be incorporated in the MTAM from which $R_{0}^{*}$ will be taken as the algorithm's input. Using Bayesian data analysis with a Markov chain Monte Carlo (MCMC) implementation, the following approach was proposed by [15]:

Step 1. Fit a MTAM and draw samples from the posterior distribution of $R_{0}^{*}$.

Step 2. Apply the IC algorithm to the posterior samples of $R_{0}^{*}$ to make the statistical decisions required. Specifically, for each query about the statistical independence between phenotypes *y_j _*and *y_j' _*(*j *≠ *j' *= 1, 2, ..., *t*) given a set of traits **S **and, implicitly, the genetic effects:

a) Obtain the posterior distribution of residual partial correlation ρ_*j*, *j'*|**S**_. These partial correlations are functions of $R_{0}^{*}$. Therefore, samples from their posterior distribution can be obtained by computing the correlation at each sample drawn from the posterior distribution of $R_{0}^{*}$.

b) Compute the highest posterior density (HPD) interval with some specified probability content for ρ_*j*, *j'*|**S**_.

c) If the HPD interval contains 0, declare ρ_*j*, *j'*|**S **_as null. Otherwise, declare *y_j _*and *y_j' _*as conditionally dependent.

Step 3. Fit a SEM using the selected causal structure (or one member within the class of statistically equivalent structures returned by the IC algorithm) as the 'true' causal structure.

More details on causal structure search based on observational data are given by [12,14]. Additionally, the approach proposed to select recursive causal structures in the quantitative genetics mixed model context is discussed by [15] and reviewed in [16].

Application of the IC algorithm involves performing a set of statistical decisions about declaring partial correlations as null or not. As the posterior distribution of these parameters becomes flatter, the statistical decisions get poorer, i.e. errors become more likely. In this scenario, using a high content HPD interval (such as 95%) protects against declaring a null correlation as non-null, but the algorithm becomes more prone to declaring non-null correlations as null. However, these two types of errors are equally important when exploring causal structure spaces [18], and therefore, in scenarios where posterior distributions of partial correlations are not sharp, results may be better when decisions are made on the basis of HPD intervals with lower content. In this article we applied several HPD content magnitudes (70, 75, 80, 85, 90, and 95%), and compared the final causal structures obtained. This approach may indicate the edges and the structures that are more stable to changes in the magnitude of HPD contents used for the statistical decisions.

### Bayesian inference and fully recursive model

The models studied were fitted via Bayesian analysis and consisted of SEM with recursive causal structures and a diagonal residual covariance matrix, as described in [15]. A fully recursive model is represented by a SEM where every entry below the diagonal of **Λ **is treated as a free parameter. The likelihood equivalence between MTAM and SEM with fully recursive causal structures [9] was explored to make inferences about the parameters of the former model by fitting the latter. The residual covariance matrix of an MTAM, which is needed for the recursive causal structure search, was obtained by fitting a fully recursive SEM and then transforming its residual covariance matrix by:

$$R_{0}^{*}={{\left(I-{\text{Λ}}_{fr}\right)}^{\prime }}^{-1}\Psi_{fr}{\left(I-{\text{Λ}}_{fr}\right)}^{-1},$$

where **Λ**_*fr *_and **Ψ**_*fr *_are, respectively, a matrix of structural coefficients and a diagonal residual covariance matrix, both associated with a fully recursive model. Such approach allowed all the models studied in this article to be fitted by using the same program.

The following joint prior distribution was assumed for location and dispersion parameters of model (1):

$$\begin{array}{lll} p\left(\text{Λ},\beta ,u,G_{0},\Psi_{0}\right) & =p\left(\text{Λ}\right)p\left(\beta \right)p\left(u|G_{0}\right)p\left(G_{0}\right)\overset{t}{\underset{j=1}{\prod }}p\left(\psi_{j}\right)\; & \\ & \propto N\left(u|0,G_{0}⊗A\right)\times IW\left(G_{0}|\upsilon_{G},G_{0}^{∙}\right)\times \; & \\ & \overset{t}{\underset{j=1}{\prod }}Inv\text{ - }\chi^{2}\left(\psi_{j}|\upsilon_{\psi },s^{2}\right),\; & \\ & \; & \end{array}$$

where *N*(**u**|**0**, **G**_0_⊗**A**) is a multivariate normal density centered at **0 **and covariance matrix **G**_0_⊗**A**, $IW\left(G_{0}|\upsilon_{G},G_{0}^{∙}\right)$ is an Inverse Wishart density with *υ*_**G **_degrees of freedom and scale matrix $G_{0}^{∙}$, *Inv*-*χ*^2^(*ψ_j_*|*υ_ψ_*, *s*^2^) is a scaled inverse-chi-square distribution with *υ_ψ _*degrees of freedom and scale parameter *s*^2^, and *ψ_j _*is the residual variance for trait *j*. Unbounded uniform distributions were assigned as prior distributions for **β **and for each structural coefficient in **Λ**. Furthermore, *υ_G_*, $G_{0}^{∙}$, *υ_ψ _*and *s*^2 ^were regarded as known hyperparameters of the prior distribution. The following hyperparameter values were used for all SEM considered: $s_{{}^{BW}}^{2}=\text{0}\text{.6, }s_{{}^{W35}}^{2}=\text{400,}$$s_{{}^{AFE}}^{2}=\text{70, }s_{{}^{AEW}}^{2}=\text{0}\text{.7, }s_{{}^{NE}}^{2}=\text{40}$ and *υ_ψ _*= 3 for every entry of the diagonal of **Ψ**; *υ*_**G **_= 7 and

$$G_{0}^{∙}=\left[\begin{array}{ccccc} 0.3 & 0 & 0 & 0 & 0\\ 0 & 200 & 0 & 0 & 0\\ 0 & 0 & 30 & 0 & 0\\ 0 & 0 & 0 & 0.3 & 0\\ 0 & 0 & 0 & 0 & 10 \end{array}\right].$$

The analyses were carried out using programs written in R [19], which are available from the authors upon request. As all fully conditional posterior distributions had closed forms, a Gibbs sampler, as discussed in [15], was applied to obtain a single chain of 300, 000 iterations for each model fitted. On the basis of visual inspection of a subset of parameters, including the structural coefficients, genetic and residual covariances, the initial 100, 000 samples of each chain were discarded as a conservative burn-in. The remaining 200, 000 iterations were regarded as samples from the posterior distributions of the parameters. The retained samples were used as basis for recursive causal structure search via IC algorithm, model comparison, and inferences about the parameters of the model fitted conditionally on the selected causal structure.

### Model comparison

Causal structures within a class of observationally equivalent structures cannot be distinguished on the basis of data evidence because they result in the same set of probabilistic conditional independencies. Therefore, they cannot be compared using criteria that rely on the likelihood function. However, structures from distinguished classes are expected to induce distinct features on the joint distribution, such that they may be compared using data evidence. In the present article, we used the Deviance Information Criterion (DIC, [20]) to compare models that present causal structures pertaining to distinct classes of structures. Such approach is followed here because different classes of causal structures may emerge from applying the search methodology using different HPD interval contents for statistical decisions. The same criterion was used to check the quality of fit of the SEM conditional on the selected causal structures by comparing them with a standard MTAM, which carries no restrictions on the dispersion parameters. Considering **θ **as a vector containing the model parameters, and D(**θ**) = -2log(*p*(**y**|**θ**)), which is called the deviance function, the DIC was obtained as follows:

$$DIC=2\bar{D}-D\left(\bar{\theta }\right),$$

where $\bar{\theta }$, which is the posterior mean of **θ**, and $\bar{D}=E_{\theta |y}D\left(\theta \right)$ were obtained from the posterior samples of **θ**.

## Results and discussion

**Figure 1:**
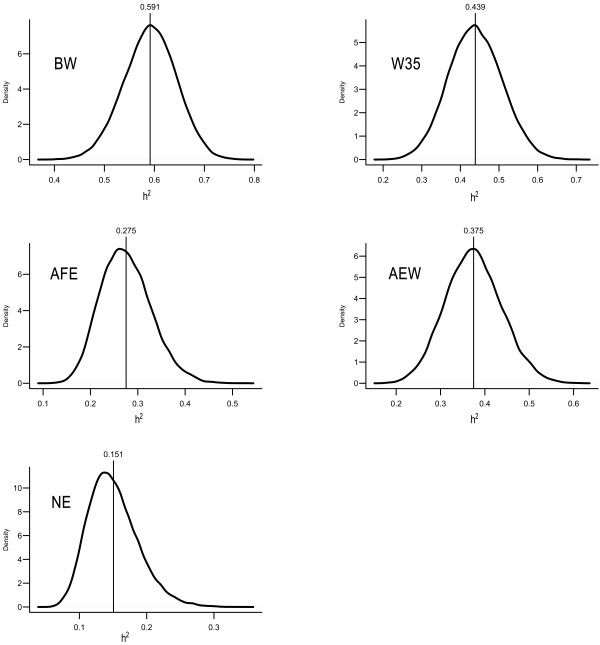
**Posterior density of MTAM heritabilities**. BW = birth weight, W35 = weight at 35 days, AFE = age at first egg, AEW = average egg weight from 77 to 110 days, and NE = number of eggs produced from 77 to 110 days.

Fitting the fully recursive SEM resulted in posterior means and 95% HPD intervals of each $R_{0}^{*}$ and $G_{0}^{∙}$ entry as given in Table 2. These matrices represent residual and additive genetic covariance matrices pertaining to a MTAM, respectively. The posterior distributions of the heritabilities as obtained from the same model are presented in Figure 1. It shows that the analyzed traits present moderate to high heritabilities, with posterior means ranging from 0.151 (NE) to 0.591 (BW).

**Table 2:** Posterior means and 95% HPD intervals for the dispersion parameters pertaining to a MTAM

**Parameter**^**a**^	Posterior mean	95% HPD Interval	**Parameter**^**a**^	Posterior mean	95% HPD Interval
$\sigma_{e_{1}}^{2}$	0.32	[0.25, 0.40]	$\sigma_{g_{1}}^{2}$	0.47	[0.36, 0.59]
$r_{e_{1}e_{2}}$	0.03	[-0.12, 0.19]	$r_{g_{1}g_{2}}$	0.41	[0.22, 0.59]
$r_{e_{1}e_{3}}$	0.07	[-0.05, 0.20]	$r_{g_{1}g_{3}}$	0.09	[-0.14, 0.31]
$r_{e_{1}e_{4}}$	-0.24	[-0.40, -0.08]	$r_{g_{1}g_{4}}$	0.64	[0.50, 0.77]
$r_{e_{1}e_{5}}$	--0.07	[-0.17, 0.04]	$r_{g_{1}g_{5}}$	0.12	[-0.14, 0.38]
$\sigma_{e_{2}}^{2}$	210.74	[164.90, 256.86]	$\sigma_{g_{2}}^{2}$	165.81	[106.42, 228.82]
$r_{e_{2}e_{3}}$	-0.13	[-0.25, -0.02]	$r_{g_{2}g_{3}}$	0.06	[-0.20, 0.31]
$r_{e_{2}e_{4}}$	0.12	[0.00, 0.25]	$r_{g_{2}g_{4}}$	0.48	[0.29, 0.67]
$r_{e_{2}e_{5}}$	0.01	[-0.09, 0.10]	$r_{g_{2}g_{5}}$	0.22	[-0.06, 0.49]
$\sigma_{e_{3}}^{2}$	35.19	[29.82, 40.53]	$\sigma_{g_{3}}^{2}$	13.42	[8.11, 19.16]
$r_{e_{3}e_{4}}$	-0.08	[-0.19, 0.03]	$r_{g_{3}g_{4}}$	0.10	[-0.16, 0.35]
$r_{e_{3}e_{5}}$	-0.12	[-0.20, -0.04]	$r_{g_{3}g_{5}}$	-0.11	[-0.40, 0.18]
$\sigma_{e_{4}}^{2}$	0.79	[0.64, 0.93]	$\sigma_{g_{4}}^{2}$	0.48	[0.30, 0.67]
$r_{e_{4}e_{5}}$	-0.01	[-0.10, 0.08]	$r_{g_{4}g_{5}}$	0.09	[-0.21, 0.37]
$\sigma_{e_{5}}^{2}$	31.02	[27.30, 34.78]	$\sigma_{g_{5}}^{2}$	5.51	[2.95, 8.31]

^a^$\sigma_{g_{j}}^{2}$ = additive genetic variance of trait *j*; $r_{g_{j}g_{j^{\prime }}}$ = additive genetic correlation between traits *j *and *j*', $\sigma_{e_{j}}^{2}$ = residual variance of trait *j*; $r_{e_{j}e_{j^{\prime }}}$ = residual correlation between traits *j *and *j*' (*j, j' *= 1 (BW), 2 (W35), 3 (AFE), 4 (AEW), 5 (NE))

**Figure 2:**
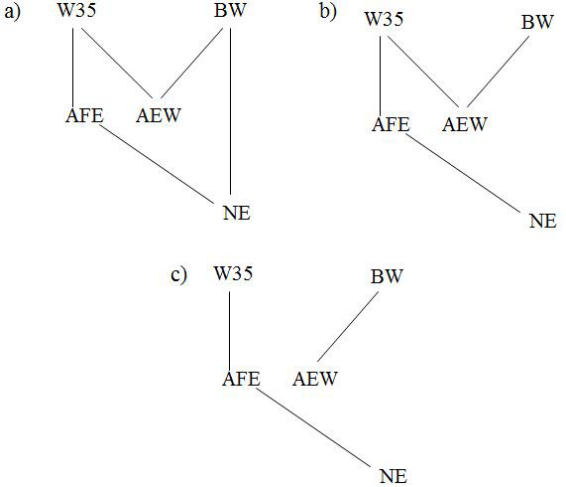
**Graphs returned by the IC algorithm using HPD 70% (a), 75, 80, 85 and 90% (b), and 95% (c) for the statistical decisions involving the traits considered**. BW = birth weight, W35 = weight at 35 days, AFE = age at first egg, AEW = average egg weight from 77 to 110 days, and NE = number of eggs produced from 77 to 110 days.

After applying the described approach for causal structure search based on different HPD interval contents, the three undirected graphs depicted in Figure 2 were selected. The output was completely undirected for each search performed because no evidence of unshielded colliders was detected. It should be stressed that finding unshielded colliders is essential for edge orienting by the IC algorithm.

**Figure 3:**
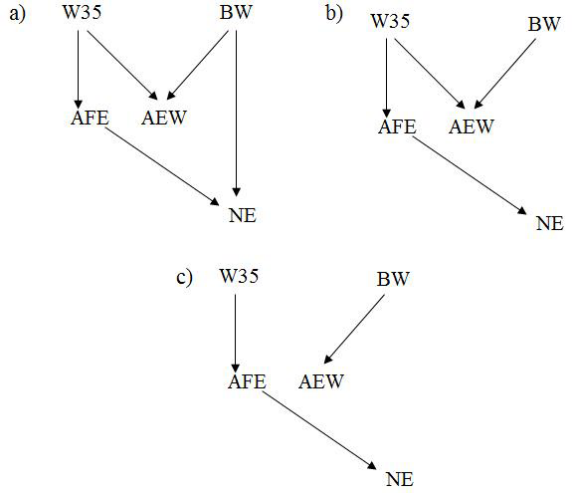
**Graphs selected by combining prior temporal information with the output of the IC algorithm using HPD 70% (a), 75, 80, 85 and 90% (b), and 95% (c) for the statistical decisions involving the traits considered**. BW = birth weight, W35 = weight at 35 days, AFE = age at first egg, AEW = average egg weight from 77 to 110 days, and NE = number of eggs produced from 77 to 110 days.

As already stated, the undirected or semidirected graphs returned by the IC algorithm represent classes of equivalent causal structures. However, the undirected graph returned when using a 70% HPD interval for the statistical decisions (Figure 2a) implies a set of observational consequences that, given the algorithm assumptions, cannot result from a SEM with recursive causal structure and independent residuals. Specifically, any attempt to direct the edges of the graph inevitably results in a causal cycle, or in unshielded colliders. Causal cycles belong to structures that are outside the explored space, and adding unshielded colliders diverges from the algorithm's output, which indicated that no evidence of such sub-structures was found from the partial correlations studied in the second step. These types of results indicate that some assumption(s) of the model or of the IC algorithm may not hold. As suggested by [12,14,18], one may combine the IC algorithm framework with prior knowledge to select causal structures. Here we choose to consider the structure in Figure 2a as a 'skeleton' and orient its edges according to temporal information. The temporal sequence followed by the phenotypic traits is: (1) BW, (2) W35, (3) AFE and (4) AEW and NE. This information prompted us to propose a causal structure as in Figure 3a, which presents two unshielded colliders that were not detected in the initial search, but carries all the edges that were previously detected.

Given the HPD contents applied to the IC algorithm, the output in Figure 2b may be considered as the most stable, since it was consistently selected when using HPD intervals of 75%, 80%, 85% and 90%. This structure is similar to the one obtained using 70% HPD intervals, except for the absence of the edge connecting BW and NE. Another difference from the previous selected structure is that this slightly sparser undirected graph reflects a set of conditional independencies that could effectively result from a recursive SEM. In other words, this undirected graph represents a non-empty class of recursive causal structures, which is in contrast to the graph previously discussed, which suggested features in the joint distribution that could not result from an acyclic SEM under the causal sufficiency assumption. However, every instance of this class conflicts with the prior knowledge regarding the temporal sequence of the studied traits, i.e. every structure of this class considers that at least one trait is affected by some other trait not yet expressed. More specifically, for every member of this causal structure class, AEW is regarded as a cause of W35, or a cause of BW, or both. Here we allowed the temporal sequence information to override the algorithm output, leading to the oriented structure presented in Figure 3b, which involves adding in the unshielded collider BW → AEW ← W35.

Finally, the last selected structure resulted from using the proposed approach based on 95% HPD intervals to make the statistical decisions. As presented in Figure 2c, this structure is also undirected, and consists of two disconnected sub-structures. Unlike the previous outputs, this class of structures carries one structure that is consistent with the temporal information regarding the studied traits, which is depicted in Figure 3c. Moreover, the edges conveyed by this undirected graph were the most stable, as they were present for every HPD interval content that was used in the search methodology.

Three distinguished SEM were constructed conditionally on the causal structures presented in Figure 3a (model A), 3b (model B) and 3c (model C). DIC's obtained for each of these models are presented in Table 3. This criterion indicated that model C, which is the simplest among these models, should be preferred. Models that present extra edges are typically expected to present a better fit. However, DIC may not assign better scores to such complex models if the extra goodness of fit achieved is not sufficient to compensate for the penalty given for model flexibility (number of parameters). Furthermore, it should be observed that models A and B carry unshielded colliders that are not supported by data evidence, i.e. the statistical consequences of their presence in the causal structure were not found when the posterior distribution of $R_{0}^{*}$ was used as input for the IC algorithm. This may have resulted in extra penalty in the DIC of these models due to decreased goodness of fit, which is suggested by their larger DIC when compared to the MTAM. On the other hand, the smaller DIC of model C when compared to MTAM's indicates that this structure is indeed plausible, presenting a good fit despite having the strongest constraints among the models studied.

**Table 3:** DIC obtained for SEM with causal structures as in Figure 3a (Model A), 3b (Model B), 3c (Model C), and for a Multiple Trait Animal Model (MTAM)

Model	DIC
A	22423.39
B	22442.31
C	22365.63
MTAM	22382.31

**Figure 4:**
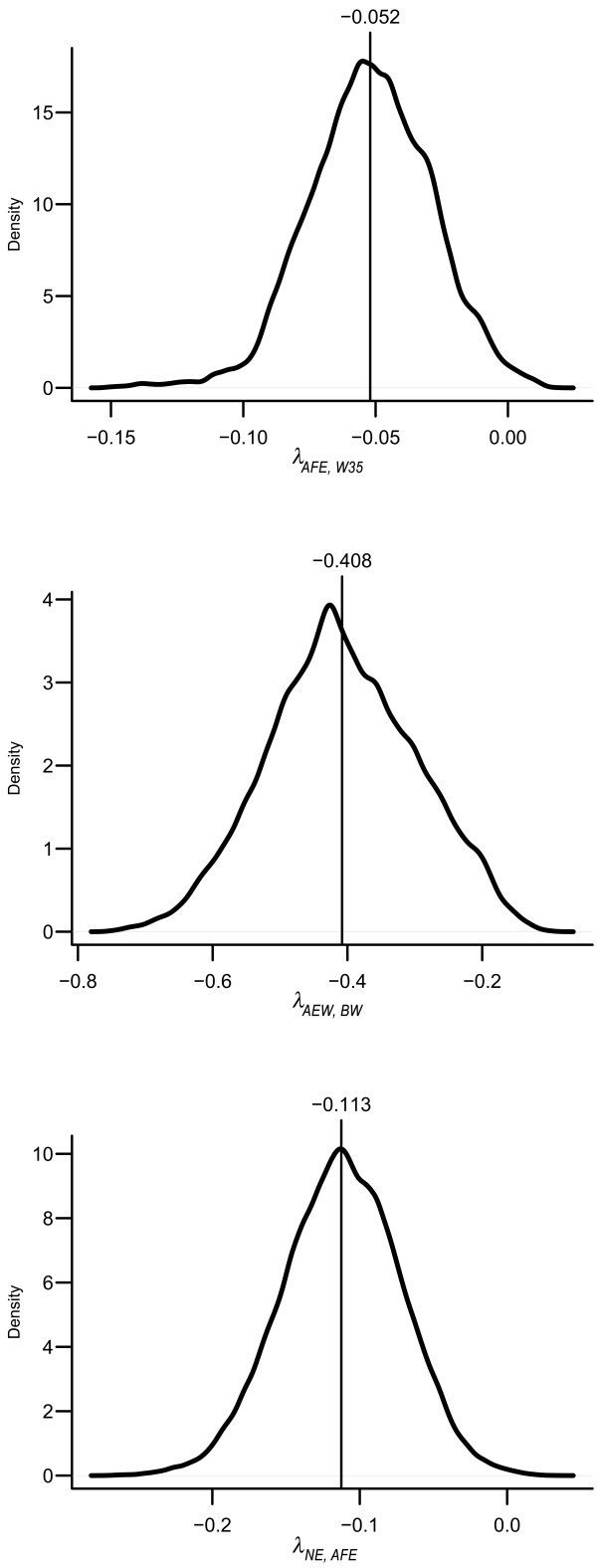
**Posterior densities of structural coefficients pertaining to Model C**.

Inferences about the dispersion parameters of a SEM that carries the selected structure (model C), as well as its structural coefficients, are presented in Table 4 and Figure 4, respectively. According to the causal structure selected and the parameter inferences, W35 imposes a negative causal effect over AFE. The posterior distribution of the magnitude of the change in AFE due to a 1g increase in W35 is given in Figure 4a, with a posterior mean of -0.052 day/g. In turn, AFE also imposes a negative effect on NE, with a posterior mean of -0.113 egg/day and the posterior distribution depicted in Figure 4c. This structure also implies that W35 presents an indirect positive causal effect on NE. Finally, inferences concerning the remaining edge indicate that BW has a negative causal effect on AEW, for which the posterior distribution is depicted in Figure 4b, with posterior mean of -0.408 g/g. At first sight, this result may seem unexpected given that phenotypes for these traits present positive covariance. However, according to the inferences for MTAM dispersion parameters, this positive phenotypic association is due to a strong positive additive genetic association (genetic covariance with posterior mean of 0.30 g^2^). Conditional on the genetic effects, the association between these traits becomes negative, as represented by residual covariance with a posterior mean of -0.12 g^2^. As a consequence, the causal association between BW and AEW could only be negative given that the causal association between BW and AEW is disconnected from the remainder of the causal structure, and given that causal sufficiency is assumed in the causal structure search.

**Table 4:** Posterior means and 95% HPD intervals for the dispersion parameters pertaining to Model C

**Parameter**^**a**^	Posterior mean	95% HPD Interval
*ψ*_1_	0.33	[0.26, 0.40]
*Ψ*_2_	195.78	[155.99, 235.96]
*ψ*_3_	34.65	[29.25, 40.03]
*ψ*_4_	0.68	[0.52, 0.84]
*ψ*_5_	30.62	[26.98, 34.32]
$\sigma_{g_{1}}^{2}$	0.45	[0.36, 0.58]
$r_{g_{1}g_{2}}$	0.45	[0.31, 0.58]
$r_{g_{1}g_{3}}$	0.21	[0.02, 0.38]
$r_{g_{1}g_{4}}$	0.80	[0.68, 0.90]
$r_{g_{1}g_{5}}$	0.07	[-0.16, 0.29]
$\sigma_{g_{2}}^{2}$	185.36	[130.23, 244.55]
$r_{g_{2}g_{3}}$	0.21	[-0.14, 0.53]
$r_{g_{2}g_{4}}$	0.58	[0.47, 0.70]
$r_{g_{2}g_{5}}$	0.19	[-0.05, 0.43]
$\sigma_{g_{3}}^{2}$	14.09	[8.34, 20.41]
$r_{g_{3}g_{4}}$	0.16	[-0.05, 0.38]
$r_{g_{3}g_{5}}$	0.09	[-0.27, 0.44]
$\sigma_{g_{4}}^{2}$	0.89	[0.51, 1.29]
$r_{g_{4}g_{5}}$	0.05	[-0.18, 0.29]
$\sigma_{g_{5}}^{2}$	5.19	[2.75, 7.87]

^a^$\sigma_{g_{j}}^{2}$ = additive genetic variance of trait *y_j_*; $r_{g_{j}g_{j^{\prime }}}$ = additive genetic correlation between traits *y_j _*and *y_j'_*, $\psi_{j}^{2}$ = residual variance of trait *y_j _*(*j, j' *= 1 (BW), 2 (W35), 3 (AFE), 4 (AEW), 5 (NE))

**Figure 5:**
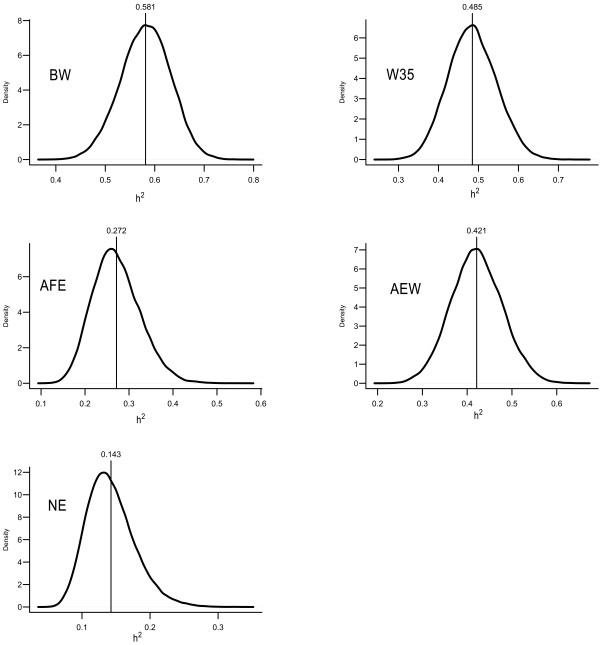
**Posterior density of heritabilities pertaining to a reduced SEM with causal structure C**. BW = birth weight, W35 = weight at 35 days, AFE = age at first egg, AEW = average egg weight from 77 to 110 days, and NE = number of eggs produced from 77 to 110 days.

The reduction of a SEM transforms model parameters in parameters of a MTAM. Inferences about heritabilities, residual and genetic covariances from a reduced model based on model C are shown in Figure 5 and Table 5. These posterior distributions are quite similar to the posterior distributions obtained for MTAM for the same parameters (Figure 1 and Table 2). This similarity was expected given that the IC algorithm searches for causal structures that are minimal and yet compatible with the distribution of the data (which is in principle described without constraints by a MTAM) and that using the chosen structure resulted in good fit according to the comparison between model C and MTAM via DIC. An opposite scenario with strong disagreements between inferences obtained under both models would indicate that features of the selected causal structure are not coherent with data evidence. This conflict would denote that the selected causal structure is not plausible.

**Table 5:** Posterior means and 95% HPD intervals for the dispersion parameters pertaining to a reduced SEM with causal structure C

**Parameter**^**a**^	Posterior mean	95% HPD Interval	**Parameter**^**a**^	Posterior mean	95% HPD Interval
$\sigma_{e_{1}}^{2}$	0.33	[0.26, 0.40]	$\sigma_{g_{1}}^{2}$	0.46	[0.36, 0.58]
$r_{e_{1}e_{2}}$	0	-	$r_{g_{1}g_{2}}$	0.45	[0.31, 0.58]
$r_{e_{1}e_{3}}$	0	-	$r_{g_{1}g_{3}}$	0.13	[-0.05, 0.30]
$r_{e_{1}e_{4}}$	-0.27	[-0.41, -0.13]	$r_{g_{1}g_{4}}$	0.64	[0.51, 0.77]
$r_{e_{1}e_{5}}$	0	-	$r_{g_{1}g_{5}}$	0.04	[-0.19, 0.27]
$\sigma_{e_{2}}^{2}$	195.78	[155.99, 235.96]	$\sigma_{g_{2}}^{2}$	185.36	[130.23, 244.55]
$r_{e_{2}e_{3}}$	-0.12	[-0.22, -0.02]	$r_{g_{2}g_{3}}$	0.02	[-0.22, 0.26]
$r_{e_{2}e_{4}}$	0	-	$r_{g_{2}g_{4}}$	0.58	[0.45, 0.70]
$r_{e_{2}e_{5}}$	0.01	[0.00, 0.03]	$r_{g_{2}g_{5}}$	0.19	[-0.05, 0.43]
$\sigma_{e_{3}}^{2}$	35.28	[29.94, 40.69]	$\sigma_{g_{3}}^{2}$	13.20	[7.91, 18.99]
$r_{e_{3}e_{4}}$	0	-	$r_{g_{3}g_{4}}$	0.02	[-0.18, 0.23]
$r_{e_{3}e_{5}}$	-0.12	[-0.20, -0.03]	$r_{g_{3}g_{5}}$	-0.12	[-0.41, 0.18]
$\sigma_{e_{4}}^{2}$	0.74	[0.61, 0.87]	$\sigma_{g_{4}}^{2}$	0.54	[0.37, 0.72]
$r_{e_{4}e_{5}}$	0	-	$r_{g_{4}g_{5}}$	0.03	[-0.21, 0.29]
$\sigma_{e_{5}}^{2}$	31.12	[27.52, 34.91]	$\sigma_{g_{5}}^{2}$	5.18	[2.81, 7.84]

^a^$\sigma_{g_{j}}^{2}$ = additive genetic variance of trait *j*; $r_{g_{j}g_{j^{\prime }}}$ = additive genetic correlation between traits *j *and *j*', $\sigma_{e_{j}}^{2}$ = residual variance of trait *j*; $r_{e_{j}e_{j^{\prime }}}$ = residual correlation between traits *j *and *j*' (*j, j' *= 1 (BW), 2 (W35), 3 (AFE), 4 (AEW), 5 (NE))

It should be stressed that one's interpretation of the output provided by the approach proposed by [15] must be guided by the (causal) assumptions one is willing to accept. This methodology could be regarded as causal structure inference in situations where the assumptions provided by [14] are accepted (namely: (1) causal sufficiency, (2) same causal relations for every individual in population, (3) faithfulness of joint distribution to an acyclic directed graph, and (4) correctness of statistical decisions). Some causal learning may still take place even if we do not accept the strong assumption of causal sufficiency (i.e., that every variable which affects two or more variables under study is already in the set of the studied variables). Applying this to the results of the present study, the existence of causal influence of AFE over NE could be claimed by simply accepting the Causal Markov Condition (which is not an assumption as strong as causal sufficiency) and by acknowledging temporal information (W35 before AFE, and the latter before NE) [21]. Nevertheless, structural equation modeling may be used without learning from the causal information carried by it. Under this circumstance, the goal may simply be to represent a joint probability distribution in a more parsimonious fashion. Generally, when a recursive causal structure is applied with this purpose, the residual covariance matrix is constructed as diagonal to achieve parameter identifiability. Nonetheless, this is exactly the statistical consequence of accepting the IC algorithm's causal sufficiency assumption, so that the described methodology may be properly used under this construction. Because the proposed approach searches for minimal causal structures, applying the retrieved structures to fit a recursive SEM would result in parsimonious modeling of joint probability distributions derived from multiple trait models.

## Competing interests

The authors declare that they have no competing interests.

## Authors' contributions

BDV, GJMR, and MAS conceived the study. MAS, RAT, and RBT were responsible for data collection and provided critical insights. BDV carried out the analysis. BDV and GJMR wrote the manuscript. All authors read and approved the final manuscript.
